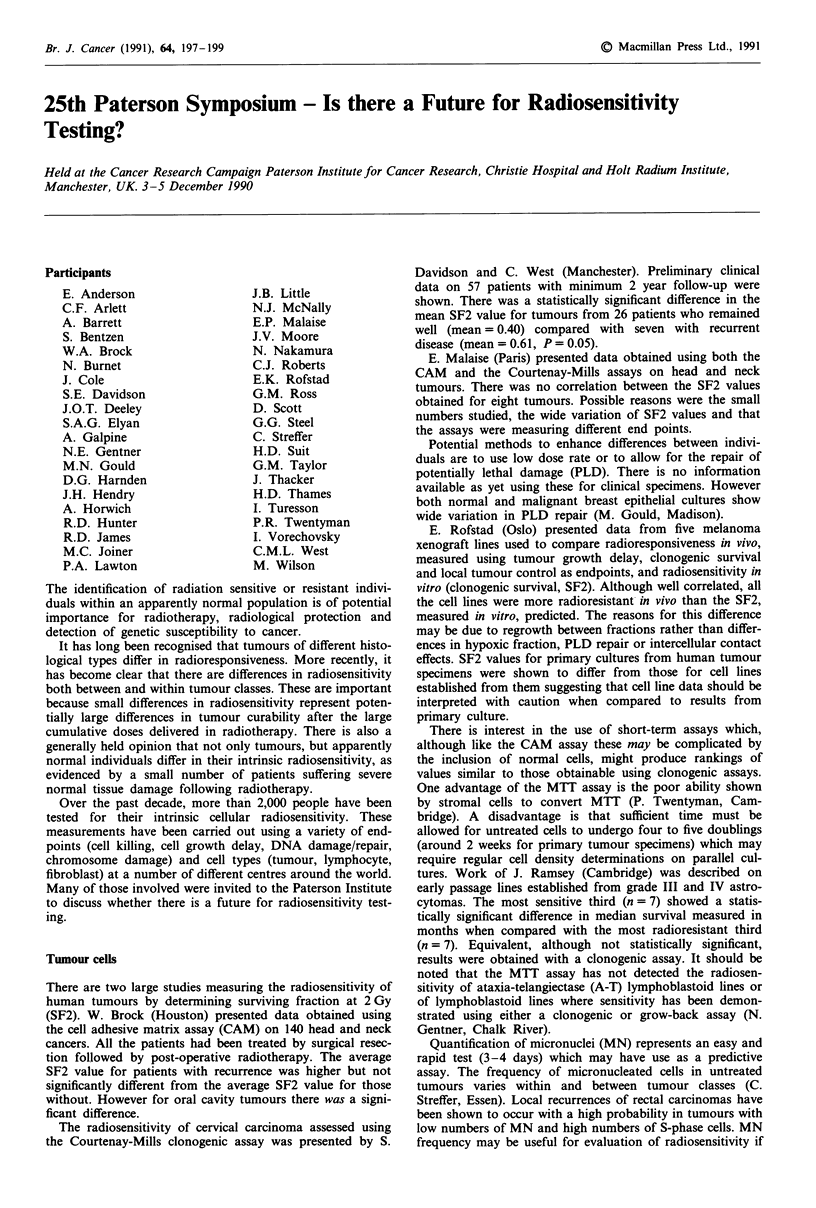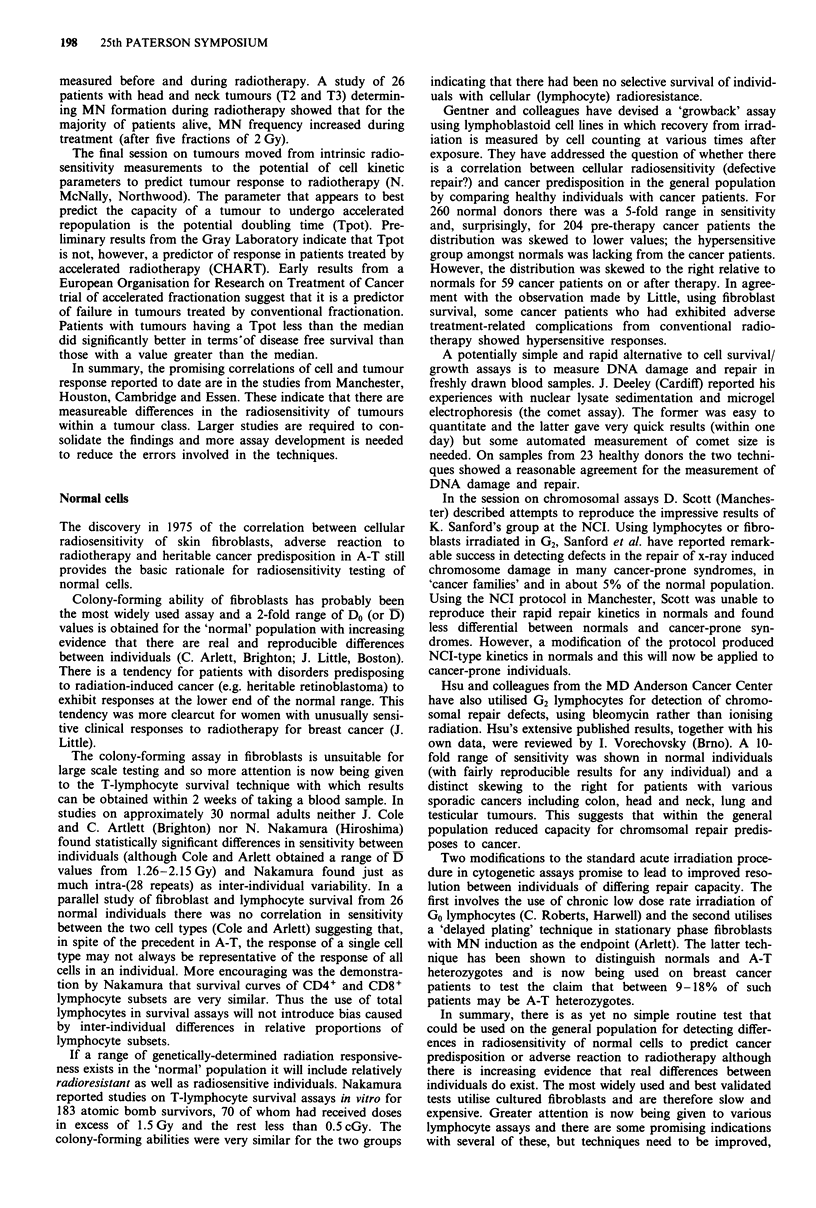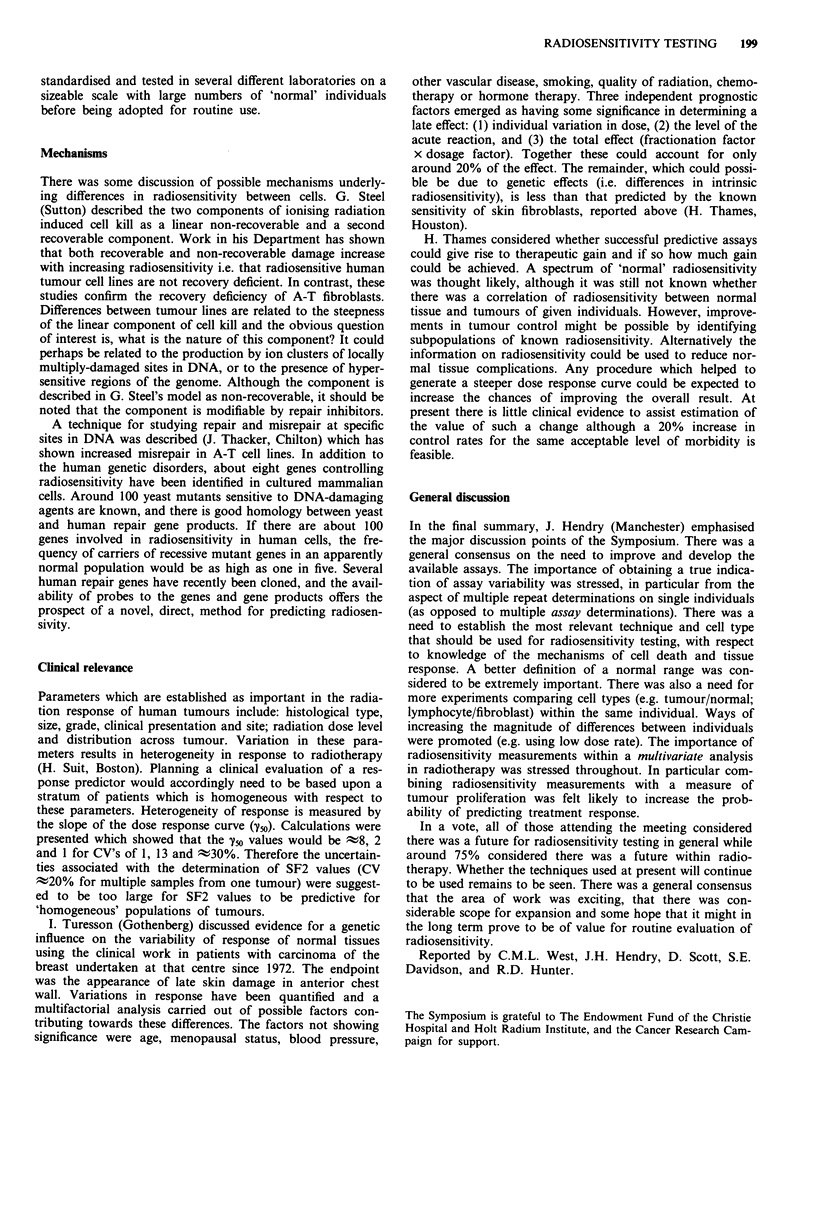# 25th Paterson Symposium--is there a future for radiosensitivity testing?

**DOI:** 10.1038/bjc.1991.270

**Published:** 1991-07

**Authors:** C. M. West, J. H. Hendry, D. Scott, S. E. Davidson, R. D. Hunter


*Br. J. Cancer* (1991), **64**, 197–199 © Macmillan Press Ltd., 1991

### 25th Paterson Symposium – Is there a Future for Radiosensitivity Testing?

*Held at the Cancer Research Campaign Paterson Institute for Cancer Research, Christie Hospital and Holt Radium Institute, Manchester, UK. 3–5 December 1990*

**Participants**

| | |
|---|---|
| E. Anderson | J.B. Little |
| C.F. Arlett | N.J. McNally |
| A. Barrett | E.P. Malaise |
| S. Bentzen | J.V. Moore |
| W.A. Brock | N. Nakamura |
| N. Burnet | C.J. Roberts |
| J. Cole | E.K. Rofstad |
| S.E. Davidson | G.M. Ross |
| J.O.T. Deeley | D. Scott |
| S.A.G. Elyan | G.G. Steel |
| A. Galpine | C. Streffer |
| N.E. Gentner | H.D. Suit |
| M.N. Gould | G.M. Taylor |
| D.G. Harnden | J. Thacker |
| J.H. Hendry | H.D. Thames |
| A. Horwich | I. Turesson |
| R.D. Hunter | P.R. Twentyman |
| R.D. James | I. Vorechovsky |
| M.C. Joiner | C.M.L. West |
| P.A. Lawton | M. Wilson |

The identification of radiation sensitive or resistant individuals within an apparently normal population is of potential importance for radiotherapy, radiological protection and detection of genetic susceptibility to cancer.

It has long been recognised that tumours of different histological types differ in radioresponsiveness. More recently, it has become clear that there are differences in radiosensitivity both between and within tumour classes. These are important because small differences in radiosensitivity represent potentially large differences in tumour curability after the large cumulative doses delivered in radiotherapy. There is also a generally held opinion that not only tumours, but apparently normal individuals differ in their intrinsic radiosensitivity, as evidenced by a small number of patients suffering severe normal tissue damage following radiotherapy.

Over the past decade, more than 2,000 people have been tested for their intrinsic cellular radiosensitivity. These measurements have been carried out using a variety of endpoints (cell killing, cell growth delay, DNA damage/repair, chromosome damage) and cell types (tumour, lymphocyte, fibroblast) at a number of different centres around the world. Many of those involved were invited to the Paterson Institute to discuss whether there is a future for radiosensitivity testing.

### Tumour cells

There are two large studies measuring the radiosensitivity of human tumours by determining surviving fraction at 2 Gy (SF2). W. Brock (Houston) presented data obtained using the cell adhesive matrix assay (CAM) on 140 head and neck cancers. All the patients had been treated by surgical resection followed by post-operative radiotherapy. The average SF2 value for patients with recurrence was higher but not significantly different from the average SF2 value for those without. However for oral cavity tumours there *was* a significant difference.

The radiosensitivity of cervical carcinoma assessed using the Courtenay-Mills clonogenic assay was presented by S. Davidson and C. West (Manchester). Preliminary clinical data on 57 patients with minimum 2 year follow-up were shown. There was a statistically significant difference in the mean SF2 value for tumours from 26 patients who remained well (mean = 0.40) compared with seven with recurrent disease (mean = 0.61, $P = 0.05$).

E. Malaise (Paris) presented data obtained using both the CAM and the Courtenay-Mills assays on head and neck tumours. There was no correlation between the SF2 values obtained for eight tumours. Possible reasons were the small numbers studied, the wide variation of SF2 values and that the assays were measuring different end points.

Potential methods to enhance differences between individuals are to use low dose rate or to allow for the repair of potentially lethal damage (PLD). There is no information available as yet using these for clinical specimens. However both normal and malignant breast epithelial cultures show wide variation in PLD repair (M. Gould, Madison).

E. Rofstad (Oslo) presented data from five melanoma xenograft lines used to compare radioresponsiveness *in vivo*, measured using tumour growth delay, clonogenic survival and local tumour control as endpoints, and radiosensitivity *in vitro* (clonogenic survival, SF2). Although well correlated, all the cell lines were more radioresistant *in vivo* than the SF2, measured *in vitro*, predicted. The reasons for this difference may be due to regrowth between fractions rather than differences in hypoxic fraction, PLD repair or intercellular contact effects. SF2 values for primary cultures from human tumour specimens were shown to differ from those for cell lines established from them suggesting that cell line data should be interpreted with caution when compared to results from primary culture.

There is interest in the use of short-term assays which, although like the CAM assay these *may* be complicated by the inclusion of normal cells, might produce rankings of values similar to those obtainable using clonogenic assays. One advantage of the MTT assay is the poor ability shown by stromal cells to convert MTT (P. Twentyman, Cambridge). A disadvantage is that sufficient time must be allowed for untreated cells to undergo four to five doublings (around 2 weeks for primary tumour specimens) which may require regular cell density determinations on parallel cultures. Work of J. Ramsey (Cambridge) was described on early passage lines established from grade III and IV astrocytomas. The most sensitive third ($n = 7$) showed a statistically significant difference in median survival measured in months when compared with the most radioresistant third ($n = 7$). Equivalent, although not statistically significant, results were obtained with a clonogenic assay. It should be noted that the MTT assay has not detected the radiosensitivity of ataxia-telangiectase (A-T) lymphoblastoid lines or of lymphoblastoid lines where sensitivity has been demonstrated using either a clonogenic or grow-back assay (N. Gentner, Chalk River).

Quantification of micronuclei (MN) represents an easy and rapid test (3–4 days) which may have use as a predictive assay. The frequency of micronucleated cells in untreated tumours varies within and between tumour classes (C. Streffer, Essen). Local recurrences of rectal carcinomas have been shown to occur with a high probability in tumours with low numbers of MN and high numbers of S-phase cells. MN frequency may be useful for evaluation of radiosensitivity if measured before and during radiotherapy. A study of 26 patients with head and neck tumours (T2 and T3) determining MN formation during radiotherapy showed that for the majority of patients alive, MN frequency increased during treatment (after five fractions of 2 Gy).

The final session on tumours moved from intrinsic radiosensitivity measurements to the potential of cell kinetic parameters to predict tumour response to radiotherapy (N. McNally, Northwood). The parameter that appears to best predict the capacity of a tumour to undergo accelerated repopulation is the potential doubling time (Tpot). Preliminary results from the Gray Laboratory indicate that Tpot is not, however, a predictor of response in patients treated by accelerated radiotherapy (CHART). Early results from a European Organisation for Research on Treatment of Cancer trial of accelerated fractionation suggest that it is a predictor of failure in tumours treated by conventional fractionation. Patients with tumours having a Tpot less than the median did significantly better in terms of disease free survival than those with a value greater than the median.

In summary, the promising correlations of cell and tumour response reported to date are in the studies from Manchester, Houston, Cambridge and Essen. These indicate that there are measureable differences in the radiosensitivity of tumours within a tumour class. Larger studies are required to consolidate the findings and more assay development is needed to reduce the errors involved in the techniques.

### Normal cells

The discovery in 1975 of the correlation between cellular radiosensitivity of skin fibroblasts, adverse reaction to radiotherapy and heritable cancer predisposition in A-T still provides the basic rationale for radiosensitivity testing of normal cells.

Colony-forming ability of fibroblasts has probably been the most widely used assay and a 2-fold range of $D_0$ (or $\bar{D}$) values is obtained for the 'normal' population with increasing evidence that there are real and reproducible differences between individuals (C. Arlett, Brighton; J. Little, Boston). There is a tendency for patients with disorders predisposing to radiation-induced cancer (e.g. heritable retinoblastoma) to exhibit responses at the lower end of the normal range. This tendency was more clearcut for women with unusually sensitive clinical responses to radiotherapy for breast cancer (J. Little).

The colony-forming assay in fibroblasts is unsuitable for large scale testing and so more attention is now being given to the T-lymphocyte survival technique with which results can be obtained within 2 weeks of taking a blood sample. In studies on approximately 30 normal adults neither J. Cole and C. Artlett (Brighton) nor N. Nakamura (Hiroshima) found statistically significant differences in sensitivity between individuals (although Cole and Arlett obtained a range of $\bar{D}$ values from 1.26–2.15 Gy) and Nakamura found just as much intra-(28 repeats) as inter-individual variability. In a parallel study of fibroblast and lymphocyte survival from 26 normal individuals there was no correlation in sensitivity between the two cell types (Cole and Arlett) suggesting that, in spite of the precedent in A-T, the response of a single cell type may not always be representative of the response of all cells in an individual. More encouraging was the demonstration by Nakamura that survival curves of $CD4^+$ and $CD8^+$ lymphocyte subsets are very similar. Thus the use of total lymphocytes in survival assays will not introduce bias caused by inter-individual differences in relative proportions of lymphocyte subsets.

If a range of genetically-determined radiation responsiveness exists in the 'normal' population it will include relatively *radioresistant* as well as radiosensitive individuals. Nakamura reported studies on T-lymphocyte survival assays *in vitro* for 183 atomic bomb survivors, 70 of whom had received doses in excess of 1.5 Gy and the rest less than 0.5 cGy. The colony-forming abilities were very similar for the two groups indicating that there had been no selective survival of individuals with cellular (lymphocyte) radioresistance.

Gentner and colleagues have devised a 'growback' assay using lymphoblastoid cell lines in which recovery from irradiation is measured by cell counting at various times after exposure. They have addressed the question of whether there is a correlation between cellular radiosensitivity (defective repair?) and cancer predisposition in the general population by comparing healthy individuals with cancer patients. For 260 normal donors there was a 5-fold range in sensitivity and, surprisingly, for 204 pre-therapy cancer patients the distribution was skewed to lower values; the hypersensitive group amongst normals was lacking from the cancer patients. However, the distribution was skewed to the right relative to normals for 59 cancer patients on or after therapy. In agreement with the observation made by Little, using fibroblast survival, some cancer patients who had exhibited adverse treatment-related complications from conventional radiotherapy showed hypersensitive responses.

A potentially simple and rapid alternative to cell survival/growth assays is to measure DNA damage and repair in freshly drawn blood samples. J. Deeley (Cardiff) reported his experiences with nuclear lysate sedimentation and microgel electrophoresis (the comet assay). The former was easy to quantitate and the latter gave very quick results (within one day) but some automated measurement of comet size is needed. On samples from 23 healthy donors the two techniques showed a reasonable agreement for the measurement of DNA damage and repair.

In the session on chromosomal assays D. Scott (Manchester) described attempts to reproduce the impressive results of K. Sanford's group at the NCI. Using lymphocytes or fibroblasts irradiated in $G_2$, Sanford *et al.* have reported remarkable success in detecting defects in the repair of x-ray induced chromosome damage in many cancer-prone syndromes, in 'cancer families' and in about 5% of the normal population. Using the NCI protocol in Manchester, Scott was unable to reproduce their rapid repair kinetics in normals and found less differential between normals and cancer-prone syndromes. However, a modification of the protocol produced NCI-type kinetics in normals and this will now be applied to cancer-prone individuals.

Hsu and colleagues from the MD Anderson Cancer Center have also utilised $G_2$ lymphocytes for detection of chromosomal repair defects, using bleomycin rather than ionising radiation. Hsu's extensive published results, together with his own data, were reviewed by I. Vorechovsky (Brno). A 10-fold range of sensitivity was shown in normal individuals (with fairly reproducible results for any individual) and a distinct skewing to the right for patients with various sporadic cancers including colon, head and neck, lung and testicular tumours. This suggests that within the general population reduced capacity for chromsomal repair predisposes to cancer.

Two modifications to the standard acute irradiation procedure in cytogenetic assays promise to lead to improved resolution between individuals of differing repair capacity. The first involves the use of chronic low dose rate irradiation of $G_0$ lymphocytes (C. Roberts, Harwell) and the second utilises a 'delayed plating' technique in stationary phase fibroblasts with MN induction as the endpoint (Arlett). The latter technique has been shown to distinguish normals and A-T heterozygotes and is now being used on breast cancer patients to test the claim that between 9–18% of such patients may be A-T heterozygotes.

In summary, there is as yet no simple routine test that could be used on the general population for detecting differences in radiosensitivity of normal cells to predict cancer predisposition or adverse reaction to radiotherapy although there is increasing evidence that real differences between individuals do exist. The most widely used and best validated tests utilise cultured fibroblasts and are therefore slow and expensive. Greater attention is now being given to various lymphocyte assays and there are some promising indications with several of these, but techniques need to be improved, standardised and tested in several different laboratories on a sizeable scale with large numbers of 'normal' individuals before being adopted for routine use.

**Mechanisms**

There was some discussion of possible mechanisms underlying differences in radiosensitivity between cells. G. Steel (Sutton) described the two components of ionising radiation induced cell kill as a linear non-recoverable and a second recoverable component. Work in his Department has shown that both recoverable and non-recoverable damage increase with increasing radiosensitivity i.e. that radiosensitive human tumour cell lines are not recovery deficient. In contrast, these studies confirm the recovery deficiency of A-T fibroblasts. Differences between tumour lines are related to the steepness of the linear component of cell kill and the obvious question of interest is, what is the nature of this component? It could perhaps be related to the production by ion clusters of locally multiply-damaged sites in DNA, or to the presence of hypersensitive regions of the genome. Although the component is described in G. Steel's model as non-recoverable, it should be noted that the component is modifiable by repair inhibitors.

A technique for studying repair and misrepair at specific sites in DNA was described (J. Thacker, Chilton) which has shown increased misrepair in A-T cell lines. In addition to the human genetic disorders, about eight genes controlling radiosensitivity have been identified in cultured mammalian cells. Around 100 yeast mutants sensitive to DNA-damaging agents are known, and there is good homology between yeast and human repair gene products. If there are about 100 genes involved in radiosensitivity in human cells, the frequency of carriers of recessive mutant genes in an apparently normal population would be as high as one in five. Several human repair genes have recently been cloned, and the availability of probes to the genes and gene products offers the prospect of a novel, direct, method for predicting radiosensitivity.

**Clinical relevance**

Parameters which are established as important in the radiation response of human tumours include: histological type, size, grade, clinical presentation and site; radiation dose level and distribution across tumour. Variation in these parameters results in heterogeneity in response to radiotherapy (H. Suit, Boston). Planning a clinical evaluation of a response predictor would accordingly need to be based upon a stratum of patients which is homogeneous with respect to these parameters. Heterogeneity of response is measured by the slope of the dose response curve ($\gamma_{50}$). Calculations were presented which showed that the $\gamma_{50}$ values would be $\approx$8, 2 and 1 for CV's of 1, 13 and $\approx$30%. Therefore the uncertainties associated with the determination of SF2 values (CV $\approx$20% for multiple samples from one tumour) were suggested to be too large for SF2 values to be predictive for 'homogeneous' populations of tumours.

I. Turesson (Gothenberg) discussed evidence for a genetic influence on the variability of response of normal tissues using the clinical work in patients with carcinoma of the breast undertaken at that centre since 1972. The endpoint was the appearance of late skin damage in anterior chest wall. Variations in response have been quantified and a multifactorial analysis carried out of possible factors contributing towards these differences. The factors not showing significance were age, menopausal status, blood pressure, other vascular disease, smoking, quality of radiation, chemotherapy or hormone therapy. Three independent prognostic factors emerged as having some significance in determining a late effect: (1) individual variation in dose, (2) the level of the acute reaction, and (3) the total effect (fractionation factor $\times$ dosage factor). Together these could account for only around 20% of the effect. The remainder, which could possible be due to genetic effects (i.e. differences in intrinsic radiosensitivity), is less than that predicted by the known sensitivity of skin fibroblasts, reported above (H. Thames, Houston).

H. Thames considered whether successful predictive assays could give rise to therapeutic gain and if so how much gain could be achieved. A spectrum of 'normal' radiosensitivity was thought likely, although it was still not known whether there was a correlation of radiosensitivity between normal tissue and tumours of given individuals. However, improvements in tumour control might be possible by identifying subpopulations of known radiosensitivity. Alternatively the information on radiosensitivity could be used to reduce normal tissue complications. Any procedure which helped to generate a steeper dose response curve could be expected to increase the chances of improving the overall result. At present there is little clinical evidence to assist estimation of the value of such a change although a 20% increase in control rates for the same acceptable level of morbidity is feasible.

**General discussion**

In the final summary, J. Hendry (Manchester) emphasised the major discussion points of the Symposium. There was a general consensus on the need to improve and develop the available assays. The importance of obtaining a true indication of assay variability was stressed, in particular from the aspect of multiple repeat determinations on single individuals (as opposed to multiple *assay* determinations). There was a need to establish the most relevant technique and cell type that should be used for radiosensitivity testing, with respect to knowledge of the mechanisms of cell death and tissue response. A better definition of a normal range was considered to be extremely important. There was also a need for more experiments comparing cell types (e.g. tumour/normal; lymphocyte/fibroblast) within the same individual. Ways of increasing the magnitude of differences between individuals were promoted (e.g. using low dose rate). The importance of radiosensitivity measurements within a *multivariate* analysis in radiotherapy was stressed throughout. In particular combining radiosensitivity measurements with a measure of tumour proliferation was felt likely to increase the probability of predicting treatment response.

In a vote, all of those attending the meeting considered there was a future for radiosensitivity testing in general while around 75% considered there was a future within radiotherapy. Whether the techniques used at present will continue to be used remains to be seen. There was a general consensus that the area of work was exciting, that there was considerable scope for expansion and some hope that it might in the long term prove to be of value for routine evaluation of radiosensitivity.

Reported by C.M.L. West, J.H. Hendry, D. Scott, S.E. Davidson, and R.D. Hunter.

The Symposium is grateful to The Endowment Fund of the Christie Hospital and Holt Radium Institute, and the Cancer Research Campaign for support.